# Novel Strategies for Developing Next-Generation Vaccines to Combat Infectious Viral Diseases

**DOI:** 10.3390/vaccines13090979

**Published:** 2025-09-17

**Authors:** Fangfeng Yuan, Martin H. Bluth

**Affiliations:** 1 Koch Institute for Integrative Cancer Research, Massachusetts Institute of Technology, Cambridge, MA 02139, USA; 2 Blood Transfusion and Donor Services, Maimonides Medical Center, Brooklyn, NY 11219, USA; 3 Department of Pathology, School of Medicine, Wayne State University, Detroit, MI 48201, USA

**Keywords:** novel vaccine, infectious viral disease, delivery system, adjuvant, imprinting, immunization

## Abstract

The development of viral vaccines faces persistent scientific and logistical challenges, particularly in the wake of the COVID-19 pandemic. This review critically examines emerging strategies to overcome key barriers in viral vaccine design and deployment. We focus on four major areas: (1) structure-guided antigen engineering to stabilize conformations; (2) the mRNA platform and its delivery system; (3) advanced adjuvant systems that enhance cellular and humoral immunity; and (4) approaches to mitigate immune imprinting and antigenic variability, such as chimeric antigens and glycan shielding. We also explore anti-idiotypic vaccination strategies and the limitations of current animal models in predicting human immune responses. In addition, to address vaccine hesitancy and inequitable access, we advocate for global collaboration in manufacturing, distribution, and public education to ensure inclusive immunization strategies. By integrating molecular insights with platform technologies, we aim to inform the rational design of future vaccines with improved efficacy and public acceptance.

## 1. Introduction

Vaccines have saved millions of lives throughout history. In general, there are two types of vaccines: live and non-live. A live vaccine can be derived from passaging a virulent strain to become less virulent while maintaining its antigenicity, or from a genetically modified strain using reverse genetics to abolish virulent factors. Both forms will replicate sufficiently in a host to activate strong protective immunity without causing symptomatic disease. Many commercial vaccines against important diseases are live, such as the oral polio vaccine, MMR (Measles, Mumps, and Rubella) vaccine, and Rotavirus vaccine [1,2]. However, safety is a major issue for live vaccines, as the virus may revert to virulence (reversion) after multiple rounds of replication in the host. Non-live vaccines come in many forms, including inactivated or killed vaccines, nucleic acid-based RNA and DNA vaccines, protein-based subunit vaccines, viral vector-based vaccines, and virus-like particles (VLPs). There are many examples of commercial non-live vaccines, such as the inactivated polio vaccine [3], protein-based hepatitis B subunit vaccine [4], VLP-based human papillomavirus (HPV) vaccine [5], and adenovirus-based rabies vaccine [6]. An adjuvant is typically needed for non-live vaccines to induce an appropriate innate immune response and improve antigen immunogenicity.

There are many effective vaccines in history. Recent vaccine development highlights diverse platforms and antigen designs for infectious viral diseases (Table 1). However, we are still facing tremendous challenges in developing effective vaccines for some infectious diseases and initiating vaccination campaigns. Scientific challenges and vaccine deployment are two major issues. For scientific challenges, the rapid mutation of viral genomes (such as HIV, hepatitis C virus, influenza virus, and SARS-CoV-2) leads to antigenic variability, which is a strategy to evade host immunity. Seasonal influenza vaccination and sequential influenza infections have led to the recall of predominantly suboptimal pre-existing immunities, known as immune imprinting, rather than effectiveness against circulating strains [7]. Thus, developing universal vaccines or employing novel strategies to overcome immune imprinting is of high interest to researchers. For some pathogens, such as African swine fever virus, the antigens inducing protective immunity have not been determined due to limited molecular understanding of host–pathogen interactions [8,9,10,11]. This uncertainty complicates the design and evaluation of subunit vaccines, but live attenuated vaccines with high efficacy have been approved. Developing vaccines that are safe and effective for immunocompromised individuals, the elderly, pregnant women, infants, and young children is particularly challenging. These populations may have different immune responses, necessitating tailored vaccine formulations and dosing schedules. For vaccine deployment challenges, access to vaccines is extremely limited in low-income regions that lack health infrastructure and financial resources. Vaccine development largely relies on commercial companies, and diseases with restricted geographical outbreaks typically do not provide commercial incentives. Additionally, anti-vaccination movements, vaccine hesitancy, lack of transparency, and limited education exist in society. The emerging mRNA delivery technology has played a critical role in developing COVID-19 vaccines [12], but its deployment also requires infrastructure for cold chain transportation. Innovative technologies are needed for easy storage and efficient mRNA delivery.

In this review, we focus on innovative strategies and the mRNA platform to address key challenges in vaccine development for infectious viral diseases. These strategies are based on advancements in novel delivery systems for mRNA vaccines, protein structure determination, novel adjuvants, and anti-idiotypic strategies. Multiple novel strategies are also discussed to overcome immune imprinting for broad protection. We also provide perspectives on developing protective vaccines by combining multiple effective approaches and emphasize the importance of global collaboration to combat viral diseases for human health.

## 2. Structure-Guided Antigen Design

The advancement of X-ray crystallography, Cryo-EM, computational biology, and technologies for neutralizing antibody isolation and mapping of antigenic sites has enabled structure-guided vaccine antigen design, focusing on protein engineering for desired, stabilized conformations [35,36]. Depending on the type of antigens to be engineered, different modifications can be made on vaccine targets, such as the introduction of proline or disulfide bonds, electrostatic and hydrogen bond substitutions, and cavity-filling. Amino acid substitution is the primary method to stabilize antigen structure.

**Introduction of proline or disulfide bonds.** Many viruses utilize class I fusion proteins to mediate fusion with the endosomal membrane and release the viral genome. After binding to receptors and proteolytic cleavage of protein subunits, the fusion part of the protein typically undergoes a conformational rearrangement to insert the fusion peptide into the cellular membrane, pulling the two membranes together for fusion. Therefore, the prefusion conformation is essential for targeting receptors. Most well-known structure-based vaccine designs focus on stabilizing proteins in the prefusion form to induce more potent neutralizing antibodies.

Two proline substitutions (2P) at the apex of the central helix and heptad repeat I can efficiently stabilize spike proteins of MERS-CoV, human coronavirus, SARS coronavirus, and SARS-CoV-2 in the prefusion conformation [12]. The 2P substitution prevents the conformational change to a coiled coil in the postfusion state and limits the fusion peptide’s release and insertion into the cellular membrane. This strategy has been proven extremely effective in inducing higher titers of neutralizing antibodies than the wild-type spike protein, ultimately leading to high protective efficacy against infection.

The inactivated RSV vaccine and postfusion F protein-based subunit vaccine elicit non- or poorly neutralizing antibody responses, resulting in minimal efficacy [37]. The resolution of prefusion and postfusion forms of the F protein allows for the identification of major antigenic sites. The highly neutralization-sensitive sites Ø and V are localized specifically on the apex of the prefusion form, whereas most of the sites (II, III, and IV) on the side and membrane-proximal region are still exposed after rearrangement in the postfusion form and only induce neutralizing antibodies with modest potency [38]. The introduction of two cysteine substitutions (S155C, S290C) stabilizes the conformation by forming a disulfide bond. This prefusion form has been proven effective in inducing neutralizing antibodies targeting prefusion-specific surfaces of the RSV F protein.

In the case of HIV, gp120 binds to cell receptors, followed by structural changes allowing gp41 to refold and fuse with the cell membrane [39]. Multiple strategies can be applied for antigen design, and the trimeric prefusion form of the envelope can be stabilized by introducing a disulfide bond between gp120 and gp41 and proline substitution in gp41 [40]. Unlike the RSV F protein, the major challenge for HIV subunit vaccine development is constant immune evasion by antigenic mutations. Therefore, structure-guided antigen design must be coupled with other novel approaches (glycan shielding, epitope scaffolding, and targeted B cell design) for HIV vaccine development.

**Cavity filling.** Particular conformations can be stabilized by filling cavities in the hydrophobic core of the vaccine antigen. An internal cavity in the protein could affect its conformation, while the introduction of amino acids with larger hydrophobic side chains could increase its stability. Two mutations in the deep pocket of the coronavirus spike RBD, I358F and F392W, fill the cavities and result in increased expression of RBD [41]. Similarly, S190F and V207L substitutions lead to cavity filling and enhanced stability of the RSV F protein [42].

**Electrostatic and hydrogen bond substitutions.** Manipulation of electrostatic interactions or hydrogen bonds also leads to increased protein stability, though with a modest contribution. Amino acid substitution of E487Q in the C terminus of the RSV F ectodomain leads to the formation of a ring of hydrogen bonds, contributing to the overall stability of the multimer [43]. The design of epitope scaffolds or chimeric proteins also allows the immune system to generate epitope-specific adaptive immunities, but this strategy has been proven less effective in inducing potent neutralizing antibodies, as protective antigens tend to induce more than one linear or conformational epitope.

Structure-guided vaccine antigen design based on the preliminary findings of MERS-CoV and HCoV spike proteins enabled the rapid development and deployment of COVID-19 vaccines [12]. Leveraging phylogenetic relationships enables the discovery of antigenic sites and mutations that confer desired conformations. To accelerate and refine this process, emerging technologies such as artificial intelligence (AI), deep learning, and high-throughput mutational scanning are increasingly being incorporated into antigen engineering workflows. AI-based modeling can predict stabilizing mutations and simulate conformational dynamics, while deep mutational scanning enables systematic evaluation of amino acid substitutions to optimize protein stability, expression, and immunogenicity. These next-generation tools offer unprecedented precision and scalability in antigen design, particularly for structurally complex or highly variable targets. Additionally, the development of a successful subunit vaccine should integrate structure-guided design with a reliable antigen delivery system and improved knowledge of B cell biology and viral pathogenesis.

## 3. mRNA Technology and Its Future

mRNA vaccines represent a rapidly advancing platform with unique advantages over traditional approaches, including the ability to generate proteins with native conformations that induce both antibody and T cell responses. Their success depends on safe and effective delivery systems, most notably lipid nanoparticles (LNPs), which protect mRNA and enhance cellular uptake. Three major formats—non-replicating linear mRNA, self-amplifying RNA (saRNA), and circular RNA (circRNA)—are under clinical investigation, each offering distinct benefits and challenges in stability, translation efficiency, and safety. Ongoing innovations focus on reducing unwanted immune activation, improving RNA purity, and achieving targeted delivery to specific tissues or immune cells. These advances are shaping the future of mRNA vaccines, broadening their potential beyond COVID-19 to diverse infectious diseases and therapeutic applications.

**Unique advantages and classification of the mRNA platform.** Common platforms used for non-live vaccine development include recombinant proteins, synthetic peptides, DNA, mRNA, viral vectors, and nanoparticles. The mRNA platform, representing an emerging platform, has several unique advantages [44]. mRNA can be directly translated into proteins in the cytosol. Compared to recombinant proteins, mRNA-translated proteins maintain their native structure and conformation, which can better induce neutralizing antibodies. Furthermore, because mRNA is translated into proteins in the cytosol, peptides derived from the protein can be presented by class I major histocompatibility complexes (MHCs) to induce CD8 T cell responses. mRNA requires a safe, stable, and effective delivery system to allow cellular uptake and release. The success of using LNPs to deliver COVID-19 mRNA has motivated broad applications of LNP-mRNA formulations [45]. LNPs have advantages over other gene and vaccine delivery technologies because they are less inflammatory, easier to manufacture, and can carry larger payloads. Furthermore, LNPs protect mRNA from host enzymatic degradation. Utilizing LNP-mRNA formulations to deliver vaccine antigens could induce both humoral and cellular immunities. Thus, we focus on the frontiers of mRNA vaccines and their delivery systems in this section.

**Figure 1:**
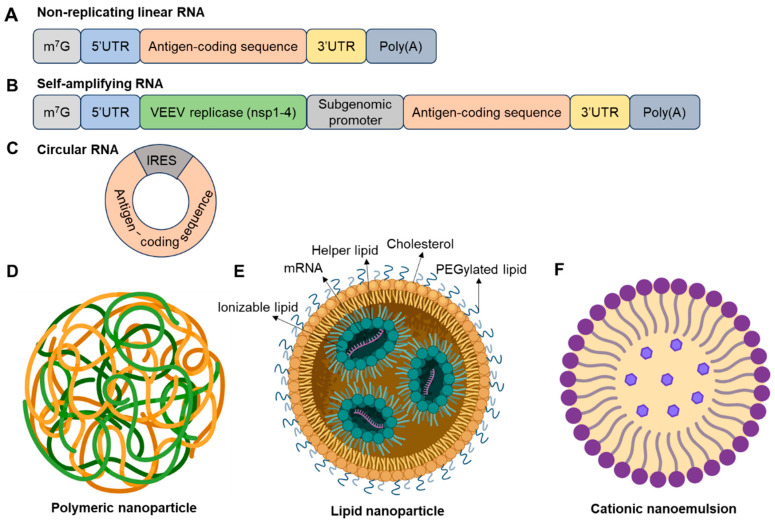
mRNA vaccine types and delivery systems. This figure illustrates various RNA formats that have been explored or utilized, including conventional mRNA (**A**), self-amplifying mRNA (**B**), and circular mRNA (**C**). Different elements of mRNA are depicted in distinct colors. For circular mRNA, untranslated regions (UTRs), can enhance protein translation. General mRNA delivery vehicles include polymers (**D**), lipid nanoparticles (**E**), and cationic nanoemulsions (**F**). The four critical lipids in lipid nanoparticles—ionizable lipid, helper lipid, PEGylated lipid, and cholesterol—are indicated. This figure for delivery systems was created using BioRender.com.

There are three forms of RNA that have entered clinical studies: traditional non-replicating linear RNA, saRNA, and circRNA (Figure 1). Non-replicating linear RNA is the most studied and has the most vaccines entering clinical trials. Key features of linear RNA include a 5′ cap, 5′-untranslated region (UTR), a protein-coding region, 3′-UTR, and a poly(A) tail. saRNA contains an additional sequence coding for RNA-dependent RNA polymerase, typically encoded by alphavirus non-structural proteins. saRNA can be given at a lower dosage compared to non-replicating mRNA due to continuous synthesis of mRNA by RNA-dependent RNA polymerase (RdRp) using the minus-sense RNA as a template. Two key issues of saRNA are the immunogenicity of introduced non-structural proteins and the limited RNA size packaged into nanoparticles. The replicase and antigens can be encoded with two separate mRNAs, which would decrease the mRNA length and increase stability but present another challenge of co-delivering two mRNAs into the same cell. Although the COVID-19 mRNA vaccines approved for massive vaccination campaigns around the globe are conventional non-replicating linear mRNA vaccines, Japan approved the first COVID-19 self-amplifying mRNA vaccine for use in adults, hoping to induce durable immunity. In contrast to linear RNAs, circular RNA is generated by linear RNA circularization and does not have a 5′ cap and 3′ poly(A) tail but contains an internal ribosome entry site [46] element to initiate translation of the antigen of interest. Recent studies have reported the inclusion of a 5′ cap into circRNA to enhance protein translation [47]. The circRNA has a closed structure connected by covalent bonds, making it presumably more resistant to degradation and potentially increasing the half-life of RNA compared to linear RNAs. The manufacturing cost for circRNA is also dramatically lower compared to linear RNAs. The major issues that limit the wide use of circRNA are low RNA circularization efficiency and a lack of safety data in humans [44]. All elements of the three types of RNAs can be fine-tuned for optimal stability, half-life, translation efficiency, and inflammatory capacity.

mRNA can be recognized by host pattern recognition receptors (PRRs), mainly Toll-like receptors (TLRs), and stimulate inflammatory cytokine production. This immune-activating capacity of mRNA can be overcome by replacing uridine with pseudouridine or its derivatives during the mRNA synthesis process in vitro [48]. Another key immunostimulatory factor is the double-stranded RNAs (dsRNAs) produced during mRNA synthesis in vitro. The reduction or elimination of dsRNAs can be achieved by engineered RNA polymerase [49], RNase treatment, or the purification of mRNA.

**Delivery systems for mRNA vaccines.** The delivery system is key for the clinical use of RNA vaccines. mRNA cannot pass through the anionic lipid bilayer of cell membranes due to its negatively charged feature and the widespread existence of nucleases [50]. Several types of nanoparticles have been developed for mRNA delivery into hosts, including lipid-based nanoparticles, polymeric nanoparticles, and cationic nanoemulsions (Figure 1 and Table 2). Lipid-based nanoparticles (LNPs) are the most widely used vehicles for mRNA delivery. LNPs contain four types of lipids (ionizable lipid, helper lipid, PEGylated lipid, and cholesterol) to encapsulate the mRNA core. The ionizable lipid (for example, SM-102, ALC-0315) is the key component of LNPs. It has a neutral pH in the bloodstream or physiological environment but becomes positively charged in the acidic environment of the endosome, promoting membrane fusion and RNA release into the cytoplasm [51]. Cholesterol promotes nanoparticle stability by filling gaps between lipids and facilitates endosomal release of mRNAs [52]. The helper lipid (for example, DSPC, DOPE) modulates surface fluidity and contributes to structural integrity [53]. An anchoring lipid (for example, DMPE, DMG) is conjugated to the hydrophilic polyethylene glycol (PEG) to form a PEGylated lipid, which stabilizes the nanoparticle and reduces non-specific interactions with macrophages [54]. In addition to lipid nanoparticles, polyplexes and polymeric nanoparticles are typically cationic and condense nucleic acids into complexes for cellular uptake. Polyethylenimine, a widely used polymer for nucleic acid delivery, demonstrates strong toxicity despite high delivery efficiency [55].

mRNA vaccines are mostly administered intramuscularly, where lipid nanoparticles are taken up by antigen-presenting cells (APCs), such as dendritic cells, through multiple entry mechanisms, such as micropinocytosis, clathrin-mediated endocytosis, and caveolae-mediated endocytosis [56]. After internalization, the ionizable lipid becomes positively charged and fuses with the endosome membrane to release mRNA molecules into the cytoplasm. Antigens of interest are translated by ribosomes through cap-dependent (linear mRNAs) or IRES-mediated (circRNAs) mechanisms. Newly synthesized proteins are transported to the proteasome for MHC class I peptide production and activation of cytotoxic T cells and [57] the lysosome for MHC class II peptide production and activation of helper T cells, which facilitate B cell survival and high-affinity antibody production. Beyond the injection site, mRNAs can also be found in the lymph nodes, spleen, liver, and blood [58].

**Table 2:** Features of key mRNA delivery systems.

Delivery System	Composition and Structure	Mechanism of Action	Encapsulation Efficiency	Targeting Capability	Immunogenicity	Biodegradability and Safety	Clinical Status (Example)	Limitations	Refs.
Lipid-Based Nanoparticles (LNPs)	Ionizable lipids, cholesterol, phospholipids, PEG lipids	Endosomal escape via pH-sensitive ionizable lipids	High (>90%)	Passive; ligand conjugation possible	Moderate; PEG-related hypersensitivity	Biodegradable; well-tolerated	Approved: SARS-CoV-2 (Pfizer-BioNTech, Moderna)	Cold chain required; reactogenicity	[44,56,59,60]
Polymeric Nanoparticles	PLGA, PEI, PBAEs, other biodegradable polymers	Proton sponge effect; slow release	Moderate (40–80%)	Tunable via surface modification	Variable; PEI may be cytotoxic	Biodegradable; potential toxicity	Phase I: Zika virus mRNA vaccine (using PBAEs)	Lower transfection efficiency; cytotoxicity risk	
Cationic Nanoemulsions	Oil-in-water emulsion with cationic surfactants	Membrane fusion; mucosal uptake	Moderate	Suitable for mucosal targeting	Moderate to high; surfactant-dependent	Limited data; surfactant toxicity possible	Preclinical: Influenza A intranasal mRNA vaccine	Stability issues; limited clinical data	

PLGA, poly(lactic-coglycolic acid); PEI, polyethylenimine; PBAE, poly(beta-amino ester).

**Safety profiles of mRNA vaccines.** Safety is currently the major challenge of mRNA vaccines. The adverse effects (also called reactogenicity) of mRNA vaccines include fever, headache, body aches, skin rash, myocarditis, and other symptoms [61,62]. Notably, these symptoms were not found in animal studies. Strong activation of the inflammatory response is often seen in vaccinated individuals. Scientists have explored the cause of reactogenicity and found that it is absent in tested animals due to interspecies differences in IL-1 receptor signaling [63]. The PEG formulated on the surface of LNPs can induce a high level of anti-PEG antibody response, which may contribute to the faster clearance of LNP-mRNAs [64]. However, the link between robust activation of PEG antibodies and vaccine reactogenicity needs to be further determined. Such studies are limited by the lack of relevant animal models. The difference in innate immune sensors between animals and humans poses challenges for reactogenicity testing in animals, let alone the various immune statuses in humans [65]. Non-human primates (NHPs) are a suitable model to assess mRNA vaccine-induced reactogenicity and immune protection, but their use faces cost and ethical concerns [66].

Proinflammatory cytokines triggered by mRNA vaccines or adjuvants of other types of vaccines could help recruit APCs to the inflammation site and promote B and T cell activation, proliferation, and differentiation into effector cells. However, an excess of inflammatory cytokines causes a cytokine storm, which manifests as fever, fatigue, etc. mRNA vaccines have faced the challenge of their highly inflammatory nature. The introduction of modified nucleosides (pseudouridine, N1-methylpseudouridine, or other nucleoside analogues) dramatically sequesters the recognition of mRNA by host immune PRRs, such as TLR2, TLR7, TLR8, and RIG-I [48]. During mRNA synthesis in vitro, T7 RNA polymerase tends to generate dsRNA that is highly immunostimulatory due to recognition by innate immune sensors. Engineering the T7 RNA polymerase to reduce the production of immunostimulatory byproducts is a hot topic in this area. Researchers have found that a double mutant polymerase (G47A +  884G) can dramatically reduce dsRNA byproducts while maintaining sufficient RNA purity and yield [49]. Another solution to this issue is through complex purification processes, such as high-performance liquid chromatography, to obtain relatively pure mRNA products.

**Targeted delivery of mRNA into a specific tissue or organ.** Another frontier of mRNA-based medicines is achieving targeted delivery to specific tissues. There are two categories of solutions to achieve targeted delivery. The first is through ligand-mediated surface modification. Various ligands (sugars, peptides, antibodies) can be added to the LNP surface to target specific tissues, organs, or tumors. For example, anisamide ligand-tethered lipidoid-based nanoparticles are reported to target fibroblasts for treating liver fibrosis [67]. Dendritic cell (DC)-targeting strategies use various ligands for specific receptors highly expressed on DCs. Sinbis virus glycoprotein, which binds specifically to DC-SIGN (a C-type lectin receptor present on the surface of both macrophages and dendritic cells) on the DC surface, can be utilized to anchor nanoparticles for targeting DCs [68]. CD40L binds to its receptor CD40 on B cells and dendritic cells. CD40L-LNPs have been used to express tumor-associated antigens (TAAs) on DCs to stimulate tumor-specific CD8 T cell responses [69]. Other examples include anti-CD19 scFv-targeting B cells [70], RGD peptides (short sequences of amino acids [arginine–glycine–aspartate] that play a crucial role in cell adhesion and signaling by interacting with integrin receptors) targeting tumor blood vessels [71], and CXCR4-targeting peptides for liver cancer [72].

The second strategy for targeting is through LNP optimizations. Different lipids exhibit distinct features. Through an ionizable lipid library screening, the iso-A11B5C1 lipid was found to selectively bind to muscle cells while showing low potency in off-target organs like the liver, spleen, and lymph nodes [73]. The charge-optimized, liposomal-formulated nanoparticles can exhibit distinct tissue tropism. The cationic lipid and RNA ratios determine the charge of the nanoparticles, which affects tissue tropism. It was found that slightly positively charged and near-neutral nanoparticles form large aggregates immediately after preparation, while positively charged liposomes primarily target the lungs rather than the spleen, and slightly negatively charged particles target the spleen exclusively [74]. To tackle the challenges of mRNA nebulization for targeted delivery into the lungs, researchers integrated multiple strategies, including optimizing the molar ratio of the three lipids (ionizable lipid, helper phospholipid, and cholesterol), adding sodium acetate in the nebulization buffer to reduce LNP aggregation, and incorporating a branched polymeric excipient (e.g., bPEG20K) [75]. These comprehensive strategies yield an optimal procedure and formulation of LNPs that exhibit improved mRNA delivery in the lungs, which could significantly advance the development of mucosal vaccines for respiratory infectious diseases, such as SARS-CoV-2, influenza virus, and respiratory syncytial virus.

In all, mRNA vaccines offer unique advantages by inducing both antibody and T cell responses, supported by lipid nanoparticle delivery systems that enhance stability and uptake. Three formats—linear mRNA, self-amplifying RNA, and circular RNA—are being explored, each with distinct strengths and limitations. Ongoing innovations aim to reduce inflammation, improve RNA stability, and enable targeted delivery, broadening mRNA vaccines’ potential beyond COVID-19.

## 4. Novel Adjuvant Development

As indispensable components of vaccines, adjuvants play an essential role in enhancing adaptive immunity and allowing lower antigen doses for increased magnitude and durability. Both immunostimulants (or danger signal molecules or PAMPs) and delivery systems can serve as adjuvants. Immunostimulants (e.g., TLR agonists) activate pattern recognition receptors (PRRs) of host cells to produce co-stimulatory cytokines, which help adaptive immune cell maturation and differentiation. Delivery systems (e.g., lipid nanoparticles) prolong antigen bioavailability inside cells, enhance APC presentation, and boost adaptive immunity. Classical adjuvants can be classified into four categories: aluminum adjuvants, emulsion adjuvants, TLR agonists, and particulate adjuvant systems [76,77].

**Aluminum and emulsion adjuvants.** Due to their well-recognized safety and reliability, aluminum adjuvants (aluminum hydroxide or aluminum phosphate) were the first adjuvants used in human vaccines for preventing and treating multiple diseases, such as tetanus, diphtheria, meningitis, and hepatitis B [78]. Aluminum adjuvants activate PRRs via DAMPs, like host DNA and uric acid, leading to IL-1β production and Th2-biased responses [79]. For emulsion adjuvants, AS03 and MF59 have been licensed for use in influenza vaccines [80]. Freund’s adjuvant, a water-in-oil emulsion, was developed by Freund and his colleagues back in the 1940s and was shown to enhance cellular and antibody response to TB [81]. The complete Freund’s adjuvant stimulates cytokine release (IL-1, IL-18, IL-33) through NOD (nucleotide-binding oligomerization domain) and other innate pathways, also favoring Th2 immunity [82]. However, due to its toxicity, Freund’s adjuvant has not been licensed for human use. In addition, the emulsion adjuvants can also promote antigen presentation by APCs through slow release and increased bioavailability.

**Novel Toll-like receptor (TLR)-based adjuvants.** TLR agonists are among the most extensively studied adjuvants, offering stronger immunostimulatory effects than classical aluminum salts. AS04, used in licensed HPV and HBV vaccines, combines aluminum hydroxide with monophosphoryl lipid A (MPLA)—a detoxified lipopolysaccharide derivative that activates TLR4 to promote Th1-biased immunity [83]. Similarly, CpG ODN (oligodeoxynucleotide) 1018, another TLR agonist, is a synthetic single-stranded DNA molecule that specifically binds to TLR-9, inducing type I interferons and proinflammatory cytokines, ultimately leading to enhanced cellular immunity [84]. Both HPV and COVID-19 vaccines evaluated in clinical trials use CpG ODN 1018 as an adjuvant [85,86]. Emerging TLR agonists, such as imidazoquinolines (R837, R848, 3M-052), activate TLR7/8 to elicit balanced humoral and cellular immunity, though formulation challenges—like systemic diffusion and antigen separation—limit their clinical translation [87]. Several alternatives to MPLA include glucopyranosyl lipid A (GLA)-stable emulsion (GLA-SE), aqueous nanosuspension (GLA-AF), and aluminum hydroxide (GLA-alum). Notably, GLA-SE is the most studied and has been used in multiple clinical trials for influenza, malaria, and HIV vaccines [88]. Lastly, double-stranded RNAs (dsRNAs) serve as adjuvants to activate TLR-3 and MDA5, producing IL-12 and type I interferons that promote cellular immune responses [77]. However, synthetic dsRNAs (poly-I:C, poly-ICLC) exhibit side effects, such as systemic fever and coagulation abnormalities.

**Particulate adjuvant.** Lastly, the particulate adjuvant system, such as AS01, contains the immunostimulant MPLA for TLR4 activation and QS-21, which activates NLRP3 for certain cytokine production (IL-1β, IL-18, IL-33) and facilitates endosomal escape for antigen presentation [89]. AS01 also induces Th1 immune responses and cytotoxic T cell responses. Licensed vaccines using AS01 include those for malaria, zoster, and tuberculosis. Although the classical adjuvant system faces issues, future adjuvant platforms should focus on increasing cellular immunity in addition to humoral responses.

**cGAS-STING (cyclic GMP-AMP synthase—stimulator of interferon genes) pathway activators.** Manganese and its derivatives activate the cGAS-STING pathway, inducing type I interferon production, promoting antigen presentation for B cell activation, and increasing CD8 cytotoxic T cell responses [90]. Manganese can also be used as a mucosal adjuvant to enhance IgA production. Another cGAS-STING pathway activator is cyclic dinucleotides (CDNs), which selectively induce strong Th1-biased immune responses [91]. CDNs are under active investigation in early-phase clinical trials, primarily in the field of cancer immunotherapy [92]. Yet, issues related to tissue targeting and stability remain key hurdles for widespread adoption.

Overall, TLR agonists remain the most popular vaccine adjuvants in preclinical and clinical trials. Incorporating multiple PRR agonists into oil-in-water emulsions may potentially enhance vaccine efficacy. The development of different vaccine types, such as routes of immunization, desired immune responses, types of pathogens, and disease stages, may require selecting the right adjuvant with desired features. Importantly, novel adjuvant development needs to combine with different delivery systems with distinct physicochemical properties for optimal vaccine efficacy.

## 5. Universal Vaccines to Overcome Imprinting

**Figure 2:**
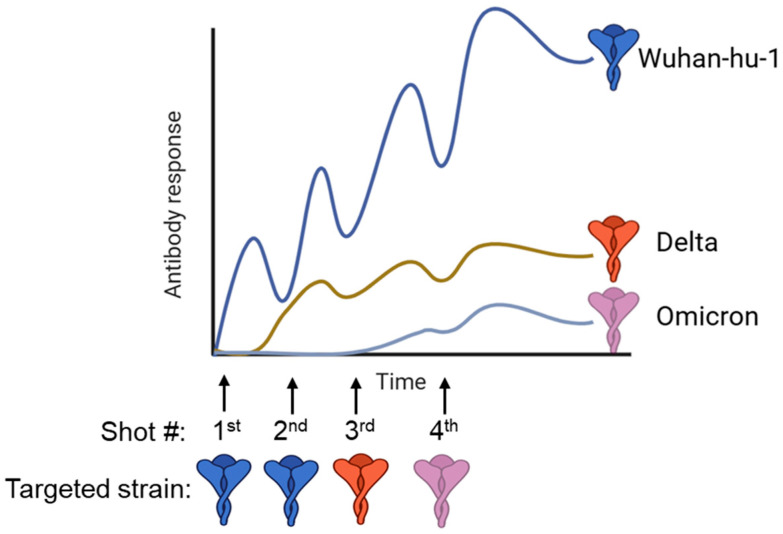
Repeated SARS-CoV-2 vaccinations elicit a dominant immune response against the ancestral Wuhan-Hu-1 strain. Variants of concern pose significant threats to global health. However, booster doses of spike-based vaccines targeting these emerging variants predominantly recall the conserved immune response imprinted by the ancestral strain, resulting in limited protection against the variants. This figure was created using BioRender. #, time of dosage.

Frequent antigenic mutations of the influenza virus and SARS-CoV-2, driven by constant selective pressure, pose significant challenges in developing universal vaccines. We have seen from COVID-19 vaccinations that boosting with a heterologous spike protein did not induce broader neutralizing antibodies against the variant; instead, the produced antibodies mostly targeted the parental strain of SARS-CoV-2 [93,94,95,96]. Immune imprinting, also called “original antigenic sin”, refers to the phenomenon where prior infections and vaccinations alter future patterns of antibody response when boosted or infected with a divergent variant strain [7] (Figure 2). During booster immunizations, immune imprinting is influenced by the competitive dynamics between memory B cells and naïve B cells [7,97,98]. Memory B cells, having higher-affinity receptors from prior exposure, expand rapidly and dominate the response, which can limit the activation of naïve B cells and shape the specificity and breadth of the resulting antibody response. Omicron breakthrough infections or boosting with Omicron antigens elicit strong anti-spike IgG responses against the Wuhan-hu-1 strain, while there is low neutralizing activity against the Omicron lineages, indicating imprinted immunity [93,94]. The COVID-19 bivalent vaccine covering BA.4/BA.5 strains elicits markedly lower neutralizing antibodies against BA.2.75.2, BQ.1.1, and XBB variants than against the Wuhan-hu-1 strain [99]. The drifted variants share multiple epitopes in the immunodominant head region, and boosting with variants recalls the memory B cells targeting conserved epitopes, resulting in robust induction of non-neutralizing antibodies against the variant, while the critical neutralizing epitopes of the variant only activate the naïve B cell repertoire. Though imprinting and antigenic variability are major issues for developing universal vaccines, several promising strategies for antigen engineering for immunization in the context of imprinting exist.

**Targeting the conserved region.** The stem region has been the target of universal influenza vaccines for inducing broadly neutralizing antibodies. A “prime and boost” strategy involves priming and boosting with chimeric antigens bearing antigenically different hemagglutinin (HA) heads but the same stem region [100]. This strategy will recall the memory B cell response for the conserved stem region upon boosting, while the antigenically different head region will only activate naïve B cells. Chimeric antigens can also be fine-tuned by replacing key antigenic sites on the head with exotic epitopes, thus providing improved functional and structural integrity [101,102]. Glycan shielding is another strategy to mask the immunodominant epitopes on the head, diverting immune activation towards broadly neutralizing epitopes in the stem region [103]. A more straightforward strategy for inducing broadly neutralizing antibody responses against the influenza virus relies solely on headless HA [104], which has been shown to protect against lethal challenges of heterosubtypic influenza viruses. Ferritin self-assembles into protein nanoparticles and can be used to display various antigens, thus forming multivalent nanoparticle vaccines. Imprints can be overcome by co-displaying heterotypic antigens on the surface of ferritin nanoparticles, which demonstrated increased breadth of protection against mismatched IAV (influenza A virus) and SARS-CoV-2 variants [105]. In silico designs via computationally optimized broadly reactive antigen (COBRA) logic contain conserved immune epitopes selected based on phylogenetic analysis and natural evolutionary pressure [106,107]. COBRA vaccine candidates enable more fine-tuned manipulations to generate desired broadly protective immune responses for influenza and COVID-19. In addition, OVX836, a recombinant nucleoprotein-based influenza A vaccine, demonstrated broad protection across multiple subtypes and achieved 80% efficacy in preclinical models [108,109]. BPL-1357, a beta-propiolactone-inactivated whole-virus vaccine targeting diverse influenza viruses (H1N9, H3N8, H5N1, and H7N3), has received FDA approval for clinical trials as an intranasal formulation [110].

**Engineering the host immune system.** Multiple strategies for manipulating the host immune system can be employed to reshape immune memory towards universal vaccine antigens. Manipulation of memory B cell clonal evolution towards immune-subdominant, broadly neutralizing epitopes can overcome prior imprints. It has been reported that memory B cell subsets that are CD80^−^PD-L2^−^-double-negative for these cell surface antigens undergo additional cycles of somatic hypermutation (SHM) and affinity maturation in the germinal center upon boosting with variants, whereas the CD80^+^PD-L2^+^ subsets differentiate into plasma cells to generate antibodies recognizing the parental strain [111]. The distinct transcription network and cytokine profiles of specific memory B cell populations can be employed to design specific drivers for desired clonal evolution. By dissecting the underlying mechanisms from a B cell perspective, broadly neutralizing antibodies targeting immune-subdominant epitopes can be produced through the selective expansion of memory B cells. In contrast to the concept of selective expansion, immunodominant B cells can also be targeted for elimination to remove the imprints and increase the breadth of the B cell response. This elimination strategy utilizes antigen-driven apoptosis during germinal center reactions. Previous studies showed that a primary immune response against heterologous antigens results in the rapid death of B cells with high affinity and avidity [112,113,114]. A similar strategy involves the passive transfer of high-affinity antibodies to compete with follicular DC-presented antigens for immunodominant B cells in an affinity-dependent manner [115]. The antibody binding with immunodominant epitopes allows B cell interaction with immune-subdominant, broadly neutralizing epitopes. Therefore, targeted elimination or suppression of immunodominant B cells through passive antibody transfer or heterologous antigen injection favors the differentiation of desired B cell subsets responsible for broad humoral immunity.

**Targeted delivery.** Lymph node or DC-targeting vaccines have become a hot research area in recent years. As the most potent and specialized APCs, DCs play key roles in the activation and regulation of innate and adaptive immunity. They patrol the environment for foreign pathogens or antigens that could activate the DC PRRs [116]. The antigen-loaded DCs prime naïve T cells into CD4^+^ T helper cells and CD8^+^ cytotoxic T lymphocytes by regulating the cytokine environment [117,118]. To enhance DC activation, the antigen delivery system can be engineered to enhance immunogenicity by carrying ligands for DC-specific surface markers (DC-SIGN, DEC-205, etc.) [119]. Poly(lactide-coglycolide) (PLG) is one of the three-dimensional biomaterial-based scaffolds that is prone to surface modification to enhance its scaffold characteristics [120]. PLG has been approved by the FDA for clinical use, and studies have shown that DC chemoattractant (GM-CSF, for example) encapsulated PLG, coupled with additional immobilization of CpG-rich oligodeoxynucleotides (CpG-ODN), a TLR9 agonist, which can attract and activate DCs in situ and induce potent host immunity against infection and cancer [120]. Hydrogel-based vaccination systems are novel injectable biomaterials and can be injected into the host and provide sustained release of SARS-CoV-2 subunits compared to PLG scaffold vaccines [121]. The DC-based vaccine is becoming an emerging era for combating infectious diseases. In addition, albumin travels specifically to the lymphoid organs and has been used in medical oncology for imaging. Vaccine antigens linked to a lipophilic albumin-binding tail demonstrated reduced systemic antigen distribution and targeted delivery into lymph nodes, where increased DC activation and T cell priming occur [122].

Developing universal vaccines for inducing broadly neutralizing antibody responses remains the focus of vaccine research. The aforementioned novel strategies to engineer antigens or the host immune system, coupled with emerging delivery systems, have the potential to overcome pre-existing immunities or immune imprints to address critical infectious viral diseases for human health.

## 6. Anti-Idiotypic Vaccination Strategies

**Figure 3:**
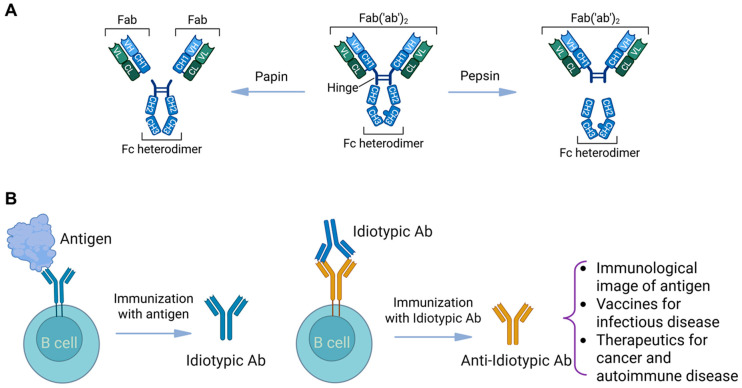
Antibody structure and idiotypic/anti-idiotypic interactions. (**A**) An immunoglobulin, with enzymes papain and pepsin and the basic structure of an antibody, showing the light chains and heavy chains. (**B**) The scheme of idiotypic/anti-idiotypic interactions and potential applications to treat various diseases. This figure was created using BioRender.

The concept that immunoglobulins themselves can serve as an immunogen to combat infectious diseases has also garnered interest. The F(ab) component of the immunoglobulin represents the “antigen binding fragment” of the antibody and comprises a variable light (VL) and variable heavy (VH) chain proximal to a constant light (CL) and constant heavy (CH) component (Figure 3A). The F(ab′)_2_ is the duplicated aspect of the antigen-binding domain and is linked to the heavy chain (IgG, IgA, IgM, IgE, IgD) via a hinge region. Enzymatic digestion with papain will cleave the antigen-binding region above the hinge region, providing two independent F(ab) components, while digestion with pepsin cleaves below the hinge region, liberating a joined F(ab′)_2_ component devoid of any heavy chain (CH2/CH3) regions. The *idiotype* represents the 3D antigen-binding cleft of the F(ab) (Figure 3B) and, if utilized as an immunogen, can propagate novel antibodies that would serve as a replica of the infectious antigen (i.e., HIV-1 gp 160 capsid protein). The F(ab) regions of these novel *anti-idiotypic* antibodies would mirror the antigen, and if used as a second immunogen, would propagate the maturation of *anti-anti-idiotypic* antibodies, which would, in essence, act as an antibody raised to the actual immunogen without ever being exposed to the actual antigen/immunogen.

It has been postulated that such anti-idiotypic antibodies may serve as a mechanism to control levels of select immunoglobulin (Ig) antibodies by neutralizing the Ig induced by their respective cytokine networks [123,124]. The production of appropriate anti-idiotypic antibodies may result in the elimination of selective Ig responses in health, whereas non-healthy (i.e., atopic, autoimmune) individuals produce the wrong type of anti-Ig antibodies, allowing unchecked deleterious (allergic, autoimmune) responses to ensue because the select Ig is not properly neutralized. Anti-idiotypic antibody networks have also been described in pregnancy to modulate HLA recognition responses and fetal viability [125,126] as well as affect organ survival in transplant recipients [127]. In addition, the interplay of antigen recognition via T (helper) and B (plasma) cell lineages has been purported to foster a perpetual wellspring of continuously stimulating anti-idiotypic inducing antibodies (serving as mimicking antigens) and maintaining the persistence of circulating anti-idiotypic antibodies for long periods of time [125]. This introduced the potential to harness anti-idiotypic antibody networks to modulate disease. To this end, anti-idiotype monoclonal antibody vaccine successes have been reported in the treatment of certain malignancies (B cell lymphoma, carcinomas). For example, vaccination with an anti-carcinoembryonic antigen (CEA) anti-idiotype antibody has led to the generation of anti-CEA antibody responses and anti-CEA proliferative T cell responses in patients with colon carcinoma [128,129].

The application of anti-idiotypic and anti-anti-idiotypic antibody responses as a means to modulate infectious diseases has also been described. Keay et al. [130] detected anti-CD4 anti-idiotype antibodies, which are antibodies that antigenically mimic HIV-1 epitopes, in sera of volunteers immunized with recombinant gp160, suggesting that molecular mimicry may enhance an immune response to the original antigen. Others have found that vaccination with the monoclonal anti-CD4 antibody, which mimics an epitope of gp120, was able to induce an immune response that inhibits gp120 binding to CD4 [131]. Furthermore, Kang et al. [132] demonstrated that primates immunized with an anti-HIV-1 anti-idiotype monoclonal antibody were able to neutralize HIV-1. Recent feline model studies have demonstrated that oral administration of anti-gp120 antibodies can stimulate mucosal and systemic immunity via anti-idiotype-mediated antibody generation, offering a novel and practical platform for HIV immunotherapy without actual antigen exposure [133].

Furthermore, anti-idiotypic antibody generation has demonstrated promising results in other animal models of infectious bacterial disease. In some of those studies, anti-idiotypic antibody vaccination was more effective than the actual antigen in inducing bactericidal antibody responses [134,135]. Anti-idiotypic antibody responses have also been proposed to affect the pathogenesis of SARS-CoV-2 infection [136].

Variations in anti-idiotypic antibody preparations may also affect their effectiveness. It is possible that “whole antibody molecule” anti-idiotypic vaccination approaches may introduce differential outcomes because of the Fc (fragment crystallizable) component influence. Each Fc component of the Ig classes has a unique binding potential with its cognate Fc receptor (FcR), which is present on different cell types. For IgG-FcγRI, FcγRIIA/B and FcγRIIIA/B are expressed on human B cells, neutrophils, eosinophil monocytes, macrophages, masts, and dendritic cells [137]; IgA-FcαRI is expressed on cells of the myeloid lineage, including neutrophils, eosinophils, monocytes, macrophages, and dendritic cells [138]; IgM-FcμR is present on T cells and, to a lesser extent, on B and NK cells among other organ tissues [139]; and IgE-Fcε R1/2 are expressed on mast cells, basophils, and other lymphocytes [140]. These Ig–receptor interactions precipitate a multitude of cytokines and other inflammatory responses that may not be ideal. To this end, enzymatic modification with papain or pepsin (see Figure 3) would allow for additional selection and purification of the F(ab) component for targeted immunization devoid of any confounding effects of the Fc region.

In contrast, it could be that the “whole antibody molecule” (Fc inclusive) anti-idiotypic vaccination approach may be advantageous since it may provide additional immunomodulatory anti-idiotypic responses. To this end, commercial intravenous immunoglobulin (IVIG) preparations have been shown to contain multiple anti-idiotypic antibodies, such as anti-factor VIII antibodies, anti-DNA autoantibodies, anti-intrinsic factor antibodies, anti-thyroglobulin (Tg) autoantibodies, anti-neutrophil cytoplasmic antibodies, anti-microsomal antibodies, anti-neuroblastoma antibodies, anti-phospholipid antibodies, anti-platelet antibodies, anti-Sm idiotypes (ID-434), and anti-GM1 antibodies. Further, IVIG has been used increasingly as an immunomodulatory agent in the treatment of autoimmune and systemic inflammatory diseases, including systemic lupus erythematosus, dermatomyositis and polymyositis, multiple sclerosis, myasthenia gravis, Guillain–Barré syndrome, and anti-phospholipid syndrome, and these beneficial clinical effects may be in part moderated by the by virtue of their whole Ig molecule anti-idiotypic neutralizing potential [141]. Dosing and immunization intervals are also likely to affect vaccination outcomes. Varying doses (ranging from 400 to 15,500 mg) of anti-idiotype antibodies produced by “rescue fusion” of malignant cells with a nonsecreting heterohybridoma (K6H6-B5) were infused three times weekly over 2 to 6 weeks in forty-five patients with non-Hodgkin lymphoma in the presence or absence of conventional immunomodulatory or chemotherapeutic agents. The majority of these patients responded to treatment, with a 66% overall and 18% complete response rate; six patients (13%) experienced prolonged complete remissions [142]. Further investigation of anti-idiotypic vaccination approaches that incorporate whole (Fc-containing) Ig molecules compared with papain/pepsin enzymatically modified F(ab) only idiotype components, their dosing intervals, the presence or absence of adjuvants, and their synergistic effects in conjunction with conventional therapy is warranted to elucidate which vaccination approaches are most ideal.

Additional concerns regarding anti-idiotypic vaccination strategies with respect to clinical feasibility, safety, and scalability are warranted. Regarding clinical feasibility, the strengths of such an immunization approach include (1) target flexibility, where antibodies can be designed against complex antigens, including haptens, poorly immunogenic molecules, or non-protein targets; (2) precision:, in which anti-idiotypic antibody maturation offers a highly specific mimicry of the original antigen, potentially enhancing immune targeting, and (3) versatility, where, in addition to anti-viral anti-idiotypic vaccination, this approach can be applied to other infectious diseases, cancers, and autoimmune conditions. However, anti-idiotypic vaccination maturation is not without challenges, including the (1) identification of functional Ab2. It is possible that only a subset of Ab2 molecules would act as true “internal image” mimics. Screening and validation would be required and can be labor-intensive. (2) The regulatory pathway involves fewer precedents existing compared to conventional vaccines, so regulatory approval may require extensive safety and efficacy studies. Clinical and preclinical applications of anti-idiotypic vaccination efficacy include trials related to malignancy in cancer. Studies by Lode et al. [143] demonstrated that the anti-GD_2_ anti-idiotype antibody, ganglidiomab, which mimics GD_2_, provided an important baseline for the development of vaccines against neuroblastoma. Studies by Ladjemi and colleagues [144] showed that an anti-idiotype-based vaccine was able to inhibit the growth of HER2-positive tumor cells in vitro and in vivo through the induction of long-lasting HER-specific immunity in a murine model of diseases. Furthermore, Wilkinson and colleagues [145] demonstrated that murine and humanized antibodies used as an anti-idiotypic vaccine were examined in mice transgenic for human MUC1—a membrane bound, polymorphic epithelial mucin expressed at the luminal surface of glandular epithelium highly expressed in an underglycosylated form on carcinomas and metastatic lesions—which were challenged by murine epithelial tumor cells transfected with human MUC1 and showed impaired tumor growth at day 35. In those studies, the highest Ab levels were found in mice that had received the addition of RAS adjuvant, suggesting that effective anti-idiotypic antibody responses may involve additional immune mechanisms. In addition, infectious disease vaccines using anti-idiotypic antibodies remain largely experimental but demonstrate proof-of-concept in preclinical models [134,135,136].

Regarding scalability, molecular biological modifications of anti-idiotypic antibodies, also known as nanobodies—which are the single domain antibody derived from the variable region of heavy chain antibody—may serve as a novel approach to maturing anti-idiotypic vaccines [146]. Nanobodies are considered superior to conventional anti-idiotypic antibody generation in that they can provide better specificity, affinity, solubility, and ability to bind antigens and can be mass produced at a lower cost [146,147]. Safety concerns will also need to be addressed. Whereas various studies have postulated the benefit of anti-idiotypic vaccination strategies as an ideal approach to treat autoimmune disease [148,149], others have cautioned that such antibodies may potentially induce autoimmune disease [134].

Thus, anti-idiotypic vaccination strategies represent a unique antigen-obviating approach to immunize healthy individuals against infectious and other diseases and provide alternatives to conventional vaccines.

## 7. New Animal Models for Vaccine Testing

Before clinical trials of a vaccine in humans, the vaccine’s safety, dose selection, immunogenicity, and efficacy need to be demonstrated using proper animal models that mimic the pathological disease of interest. Although obtaining an ideal animal model for human vaccine evaluation is challenging, it should be economical, readily obtainable, and well-recapitulate the human immune response and disease features.

**Existing models for COVID-19.** Numerous animal models have been established for SARS-CoV-2 studies on pathogenesis and vaccine testing. Non-human primates (Rhesus macaques, African green monkeys, baboons, etc.) represent the ideal model due to their greatest similarity to humans in many aspects (immune system, physiological characteristics, etc.), but they are typically expensive [150]. Alternative models for SARS-CoV-2 have been examined extensively. SARS-CoV-2 can infect a broad range of animal species (tigers, deer, cats, dogs, etc.) with varied disease manifestations [151]. As the most widely used experimental animal, mice have been engineered to study viral pathogenesis and vaccines. Wild-type mice are not susceptible to SARS-CoV-2 infection due to the absence of the human ACE2 (hACE2) receptor. Genetically modified mice, established through random insertion or precision knock-in of hACE2, demonstrate distinct clinical manifestations depending on the hACE2 delivery technology used (e.g., CRISPR gene editing, adeno or adeno-associated viral transduction, etc.) [152]. In contrast to the upper respiratory infection of COVID-19 in humans, genetically modified mice exhibit obvious weight loss, respiratory distress, and impaired lung function. SARS-CoV-2 infection in hACE2 transgenic mice shows lung pathology similar to human pneumonia in severe infection cases [153,154]. Mouse-adapted SARS-CoV-2 strains can be used for pathogenesis studies in mice [155,156]. Different genetic backgrounds of mice induce distinct types of immune responses, as BALB/c mice tend to activate Th2 immunity while C57BL/6 mice activate more Th1 immunity [157,158]. Generally, viruses may use multiple receptors for entry, so transgenic mice with a single receptor gene do not reflect real-world infection.

**Limitations of animal models.** Animal models are essential for preclinical vaccine testing but have inherent limitations. Differences in immune systems and genetic backgrounds can lead to responses that do not fully recapitulate human immunity [159,160,161,162]. For example, transgenic mice expressing a single human receptor may not capture the complexity of human viral entry and immune interactions. Different species (mice, ferrets, non-human primates) and even strains within a species can show distinct susceptibility, immune responses, and disease manifestations. Many models fail to reproduce the full spectrum of disease severity, long-term complications, or population-specific responses, such as those in aged, pregnant, or immunocompromised individuals. Additionally, practical constraints, including ethical considerations, cost, and reliance on engineered or artificial systems, can limit their predictive value for human vaccine efficacy and safety.

**Future directions.** Future development of animal models should address specific disease issues. A mouse model mimicking more severe disease is needed for COVID-19 studies. Animal models that support viral persistence or long COVID are not readily available. Animal models reflecting pathogenesis in aged groups, pregnant women, young children, and immunosuppressive groups are especially urgently needed [163]. Precision gene editing provides a solution for controlled manipulation of the mouse genome compared to random insertions. Genetic knock-in technologies make it easier to establish animal models reflecting the human immune system for vaccine testing. Overall, mice are the most convenient animal model to modify for replicating disease progression and clinical features of pathogenic agents.

## 8. Global Collaboration and Education

Low vaccine accessibility and the anti-vaccine movement are partially due to low compliance. For example, during the early stages of the COVID-19 pandemic, the acceptance rate for the vaccine ranged from 56% to 75%, which is insufficient to reach the herd immunity threshold of 80% or higher [164,165,166]. The acceptance rate for booster vaccines targeting emerging variants is much lower, leading to a dramatic decrease in vaccine revenue for Moderna and Pfizer [167]. To improve patient compliance with vaccinations, novel vaccination methods are being developed to replace traditional needles and syringes. Immunization without needles or syringes would be more accepted, safer, and suitable for rapid mass use in the case of a disease pandemic [168]. This practice is critical for both developed and developing countries to expand immunization coverage.

Needle-free technology can be classified into two types of delivery modes: mucosal surface and skin. For mucosal immunization, multiple strategies have been developed to overcome the mucosal barrier for vaccine delivery to specialized microfold (M) cells. The oral polio vaccine, developed decades ago, sets the standard for ease of administration, and these types of vaccines are generally live attenuated viruses with tissue tropism for mucosal areas [169]. Similarly, nasal vaccines for influenza virus and SARS-CoV-2 have been developed using live attenuated viruses or adenoviral vectored platforms [170,171,172]. In addition to these virus-based vaccines, novel strategies have been developed to deliver mRNAs to mucosal lymphoid tissues using nanoparticles or synthetic biology tools. Combining novel intranasal adjuvants to overcome mucosal barriers, lipid nanoparticles, or polymers can be designed with optimal tissue-targeting lipids and compositions to encapsulate mRNA for targeted and efficient mucosal delivery [75,86].

The other delivery mode for needle-free technology is through the skin, which typically contains a rich network of immune cells, particularly Langerhans cells as antigen-presenting cells (APCs). Jet injectors are needle-free instruments that inject vaccines transcutaneously or subcutaneously through a minute orifice under high pressure [173,174]. Single-dose jet injectors using disposable cartridges and nozzles have been developed for mass immunizations [175,176]. Microneedles are another novel vaccine delivery system that integrates biodegradable or food-grade self-assembling materials with 3D printing technology to manufacture patches containing arrays of microneedles [177,178,179,180]. The microneedles range from 50 to 900 µm in length and can efficiently inject and slowly release vaccine antigens or nucleic acids (DNA or RNA) into the skin. Microneedles are a very promising delivery technology, though issues like instability and dosage inadequacy need to be addressed. Overall, needle-free technologies have emerged as novel delivery systems offering a painless and accepted means for mass immunizations. Additionally, novel delivery platforms that eliminate the need for a cold chain would improve infrastructure and increase accessibility to vaccines.

To reduce sociopolitical barriers to vaccination, the scientific community needs to provide more education to the general public and political figures about the existing knowledge and importance of vaccines. Being transparent with the non-scientific community is important, as vaccine effectiveness can be affected by multiple factors, such as age, immune status, adherence to schedule, and the disease itself [181,182]. Developing infectious disease vaccines is mainly driven by biotech and biopharma companies for profit. However, human diseases without an epidemic or pandemic can make companies less likely to develop vaccines. Thus, the role of government is critical in providing incentives for vaccine development. The COVID-19 pandemic has shown that government incentives can ensure that mRNA vaccines from Moderna and Pfizer are developed at unprecedented speeds, though there are also commercial interests for companies. There is still a long way to go in terms of reducing vaccine skepticism, especially among political figures who authorize government funding for the scientific community. What the scientific community can do is develop novel technologies, such as nanomedicine, and improve vaccine efficacy. However, novel vaccine platforms generally require established policies by regulatory agencies. For a streamlined regulatory approval process, policymakers need to establish comprehensive guidelines to evaluate novel platform properties, such as stability, safety, aggregation potential, immunogenicity, and efficacy. Nucleic acid-based vaccines pose potential risks of modifying the host genome or germline, which presents ethical and regulatory challenges. Preclinical and clinical vaccine trials will need to inform patients about essential information regarding potential risks. To increase vaccine accessibility through international collaboration, organizations such as the WHO (World Health Organization), CEPI (Coalition for Epidemic Preparedness Innovations), and Gavi (a Vaccine Alliance organization) can provide platforms for cross-border collaboration, pooling resources for vaccine development, procurement, and distribution, especially in low- and middle-income countries. International data sharing frameworks and joint clinical trial networks can also accelerate evaluation and approval processes across regulatory agencies, reducing duplication and ensuring equitable access.

## 9. Conclusions

Developing next-generation vaccines that are safe, efficacious, cost-effective, and easily deployable requires collaboration between multidisciplinary teams, including material engineering, structural biology, computational biology, immunology, bioinformatics, and policymakers. Novel delivery platforms, such as LNP-mRNA technology, induce robust adaptive immune responses. Structure-guided antigen design provides a more rational approach to potentiating protective immunity. Novel adjuvants will promote the activation of adaptive immunity. Developing universal vaccines to overcome the imprinting issue requires advanced antigen design and an understanding of virus–host immune interactions. Anti-idiotypic vaccination strategies provide the opportunity to harness the power of the immune system itself as a mode of immunization. Novel animal model development would facilitate the evaluation of vaccine immunogenicity and efficacy, providing robust evidence before preclinical and clinical testing in humans. Vaccine skepticism and hesitation can be overcome using combined strategies, including improved infrastructure, public education, technological advancement, and established regulatory policies. All these elements constitute a comprehensive strategy to combat infectious diseases and improve human health.

## Figures and Tables

**Table 1:** Recent advances in viral vaccine development.

Vaccine Candidate	Target Virus	Platform	Route	Antigen Design	Clinical Trial Phase and ID	Unique Design Feature	Refs.
SteMos1 (NIAID)	Influenza	Nanoparticle (HA stem)	IM	Structure-guided HA stem + ALFQ adjuvant	Phase I: NCT07111078	HA stem-only nanoparticle for universal flu vaccine	[13,14]
OVX835 (Osivax)	Influenza	Recombinant NP antigen	IM	Conserved nucleoprotein (NP)	Phase 2a: NCT04192500	T cell-focused design targeting internal antigen	[15,16]
DentalFloss-M2e	Influenza	Gold nanoparticle	Floss-based	M2e peptide scaffold	N/A	Floss-like scaffold for mucosal delivery	[17]
cHA-ΔNS1-LAIV (CIVICs)	Influenza	Live attenuated vaccine	IN	Chimeric HA + NS1 deletion	N/A	NS1 deletion enhances safety and mucosal immunity	[18]
GammaFlu (Gamma Vaccines)	Influenza	Whole-virus inactivated	IM	Broad-spectrum antigen mix	N/A	Self-adjuvanting	[19]
RSM2eFP (CAS)	Influenza	Bacillus subtilis spore-based oral vaccine	Oral	M2e + fusion peptide	N/A	Thermostable spore-based oral delivery	[20]
mRNA-1010 (Moderna)	Influenza	mRNA-LNP	IM	HA antigens from 4 strains	Phase I/II: NCT04956575	Quadrivalent seasonal mRNA flu vaccine	[21,22]
ARCoV (Walvax)	SARS-CoV-2	mRNA-LNP	IM	RBD domain	Phase III: NCT04847102	RBD-only design for thermostability	[23]
SAM-COVID (Gritstone)	SARS-CoV-2	Self-amplifying mRNA	IM	Spike + T cell epitopes	Phase I: NCT04776317	Self-replicating RNA for dose-sparing	[24]
ABNCoV2 (AdaptVac/Bavarian Nordic)	SARS-CoV-2	VLP-mRNA hybrid	IM	RBD displayed on VLP	Phase I: NCT04839146	Capsid VLP display enhances B cell activation	[25]
mRNA-1073 (Moderna)	SARS-CoV-2 + Influenza	mRNA-LNP	IM	Spike + HA antigens	Phase I: NCT05585632	Dual-pathogen respiratory vaccine	[26]
UB-612 (Vaxxinity)	SARS-CoV-2	Peptide-based subunit	IM	RBD + T cell epitopes	Phase III: NCT05293665	Synthetic peptide for T cell targets	[27]
mRNA-1345 (Moderna)	RSV	mRNA-LNP	IM	prefusion F protein	Phase I: NCT04528719	Structure-guided prefusion F design	[28]
ChAdOx1 RSV (Oxford)	RSV	Adenoviral vector	IM	Prefusion F protein	Phase I: NCT04754776	ChAdOx1 vector with stabilized RSV antigen	[29]
DS-Cav1 (NIH)	RSV	Protein subunit	IM	prefusion F protein	Phase I: NCT03049488	First rationally engineered RSV antigen	[30]
mRNA-1083 (Moderna)	RSV + SARS-CoV-2	mRNA-LNP	IM	Spike + RSV F protein	Phase III: NCT05827926	Dual-pathogen mRNA respiratory vaccine	[31]
mRNA-1653 (Moderna)	hMPV + PIV3	mRNA-LNP	IM	Engineered fusion proteins	Phase I: NCT04144348	Combined pediatric respiratory vaccine	[32]
mRNA-1893 (Moderna)	Zika virus	mRNA-LNP	IM	Zika envelope protein	Phase II: NCT04917861	mRNA-encoding E protein	[33]
CV7202 (CureVac)	Rabies virus	Protamine-mRNA	IM	Rabies glycoprotein	Phase I: NCT03713086	Protamine-complexed mRNA for enhanced stability	[34]

IM, intramuscular; IN, intranasal; RSV, respiratory syncytial virus; hMPV, Human Metapneumovirus; PIV3, Parainfluenza Virus Type 3; VLP, virus-like particle; RABV-G, Rabies Virus Glycoprotein; N/A, not applicable.
